# Anti-Proliferation Activity of a Decapeptide from *Perinereies aibuhitensis* toward Human Lung Cancer H1299 Cells

**DOI:** 10.3390/md17020122

**Published:** 2019-02-18

**Authors:** Shuoqi Jiang, Yinglu Jia, Yunping Tang, Die Zheng, Xingbiao Han, Fangmiao Yu, Yan Chen, Fangfang Huang, Zuisu Yang, Guofang Ding

**Affiliations:** 1 Zhejiang Provincial Engineering Technology Research Center of Marine Biomedical Products, School of Food and Pharmacy, Zhejiang Ocean University, Zhoushan 316022, China; jsq_sxty@163.com (S.J.); lxj19950329@163.com (Y.J.); yangzs87@163.com (D.Z.); 17805805851@163.com (X.H.); fmyu@zjou.edu.cn (F.Y.); cyancy@zjou.edu.cn (Y.C.); gracegang@126.com (F.H.); abc1967@126.com (Z.Y.); 2 Laboratory of Aquatic Products Processing and Quality Safety, Zhejiang Marine Fisheries Research Institution, Zhoushan 316021, China

**Keywords:** *Perinereis aibuhitensis*, decapeptide, lung cancer, cell proliferation, apoptosis

## Abstract

*Perinereis aibuhitensis* peptide (PAP) is a decapeptide (Ile-Glu-Pro-Gly-Thr-Val-Gly-Met-Met-Phe, IEPGTVGMMF) with anticancer activity that was purified from an enzymatic hydrolysate of *Perinereis aibuhitensis*. In the present study, the anticancer effect of PAP on H1299 cell proliferation was investigated. Our results showed that PAP promoted apoptosis and inhibited the proliferation of H1299 cells in a time- and dose-dependent manner. When the PAP concentration reached 0.92 mM, more than 95% of treated cells died after 72 h of treatment. Changes in cell morphology were further analyzed using an inverted microscope and AO/EB staining and flow cytometry was adopted for detecting apoptosis and cell cycle phase. The results showed that the early and late apoptosis rates of H1299 cells increased significantly after treatment with PAP and the total apoptosis rate was significantly higher than that of the control group. Moreover, after treatment with PAP, the number of cells in the S phase of cells was significantly reduced and the ability for the cells to proliferate was also reduced. H1299 cells were arrested in the G2/M phase and cell cycle progression was inhibited. Furthermore, the results of western blotting showed that nm23-H1 and vascular endothelial growth factor (VEGF) protein levels decreased in a dose-dependent manner, while the pro-apoptotic protein and anti-apoptotic protein ratios and the level of apoptosis-related caspase protein increased in a dose-dependent manner. In conclusion, our results indicated that PAP, as a natural marine bioactive substance, inhibited proliferation and induced apoptosis of human lung cancer H1299 cells. PAP is likely to be exploited as the functional food or adjuvant that may be used for prevention or treatment of human non-small cell lung cancer in the future.

## 1. Introduction

Primary bronchogenic carcinoma is referred to as lung cancer and can occur at various levels of the bronchial epithelium or glands in the form of a malignant tumor [1]. The incidence and mortality of lung cancer have increased year by year and lung cancer is a disease that seriously threatens human health and life. According to statistics from the United Nations and the European Union, the lung cancer is the most common cause of cancer death, with an estimated 388,000 lung cancer cases resulting in death in Europe in 2018 [2]. In addition, according to the American Cancer Society’s 2018 report, the numbers of new cancer diagnoses and cancer deaths in the United States in 2018 are estimated to be 234,030 and 154,050, respectively, with lung cancer having the highest mortality rate in both sexes [3]. According to the pathological morphology, lung cancer generally can be classified into two categories: small-cell lung cancer (SCLC) and non-small-cell lung cancer (NSCLC). NSCLC represents 80% to 85% of all lung cancers and the early diagnosis rate is low. About 85% of patients have distant metastases or inoperable tumors at the time of diagnosis, leaving only comprehensive chemotherapy-based treatment [4]. However, due to the poor sensitivity of NSCLC to chemotherapy and the lack of ideal selective agents, toxicity and drug resistance to existing chemotherapy drugs, the dose of antitumor drugs is limited, the efficacy is affected, and treatment can fail.

Peptides are important natural products in various organisms and their broad spectrum of activities promotes the completion of various complex physiological functions in the body. The requirements for life in the ocean cause marine organisms to produce and accumulate a large number of peptides with unique structures and special biological activities. Extensive research has found that some marine peptides or their derivatives have potential as nutrient supplements or have medicinal value, such as antimicrobial peptides [5], antiviral peptides [6], antitumor/cytotoxic peptides [7], angiotensin-I-converting enzyme (ACE) inhibitory peptides [8], and antioxidant peptides [9,10]. Many of these have been developed as pharmaceutical or dietary supplements. Recently, many polypeptide fractions were isolated from marine organisms with potent anti-proliferative activity against cancer cells. For example, Yu et al. [11] demonstrated that a pentapeptide (Ile-Leu-Tyr-Met-Pro, ILYMP) isolated from *Cyclina sinensis* protein hydrolysate could induce apoptosis in DU-145 prostate cancer cells, whereas Wu et al. [12] reported that an oligopeptide (Tyr-Val-Pro-Gly-Pro, YVPGP) from *Anthopleura anjunae* showed anticancer activity against DU-145 prostate cancer cells. Additionally, Wang and Zhang [13] found an anti-proliferative peptide isolated from the protein hydrolysate of *Spirulina platensis* that exhibited strong inhibition on HT-29 cancer cells with the half maximal inhibitory concentration (IC_50_) of 99.88 µg/mL. Similarly, Pan et al. [14] purified a hexapeptide (Phe-Ile-Met-Gly-Pro-Tyr, FIMGPY) from the protein hydrolysate of skate (*Raja porosa*) cartilage, which exhibited dose-dependent anti-proliferative activity with an IC_50_ of 4.81 mg/mL against the HeLa cells.

*Perinereis aibuhitensis* belongs to the *Annelida*, *Polychaeta*, *Errantia*, *Nereidae, Nereis* genus [15]. It is very common in rocky shores, rocks, algal cones and coral reefs or soft sediments in intertidal zones. In recent years, due to the economics of *Perinereis aibuhitensis* rearing, it has provided abundant materials for pharmacological research. It has been reported that Nereis extract shows good insecticidal [16], antithrombotic [17], antihypertensive [18] and antimicrobial [19] activities. However, there are few reports on the inhibitory effects of functional peptides from *Perinereis aibuhitensis* on human lung cancer H1299 cells. *Perinereis aibuhitensis* peptide (PAP) is a decapeptide (Ile-Glu-Pro-Gly-Thr-Val-Gly-Met-Met-Phe, IEPGTVGMMF) with anti-cancer activity that is purified from an enzymatic hydrolysate of *Perinereis aibuhitensis* in our previous study [20]. However, the mechanism of its anticancer activity was not well illustrated. In this study, in vitro cultured human lung cancer H1299 cells were used to observe the effect of PAP on tumor cell proliferation, apoptosis and metastasis, which may lead to another alternative high value-added utilization of *Perinereis aibuhitensis*.

## 2. Results and Discussion

### 2.1. Effects of PAP on the Proliferation of H1299 Cells

**Figure 1:**
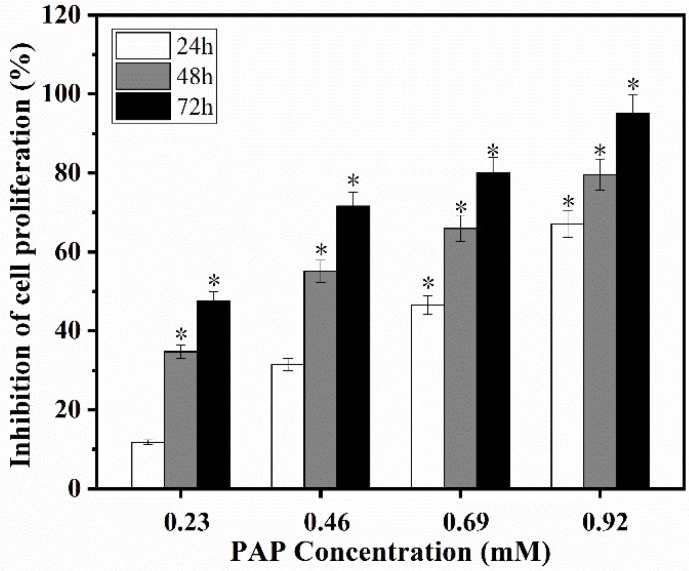
The inhibitory effect of *Perinereis aibuhitensis* peptide (PAP) on the proliferation of H1299 cells. H1299 cells were treated with different concentrations of PAP for 24, 48 and 72 h. All data are presented as the mean ± standard deviation (SD) of three experiments. (*) Results are significantly different from the control (*p* < 0.05).

Normal human cells are tightly regulated, but excessive proliferation of cells and uncontrolled regulation of apoptosis can lead to uncontrolled growth of tumors. The inhibitory effect of PAP on the proliferation of H1299 cells was determined using the CCK-8 method. There was a dose-dependent inhibition of H1299 cell proliferation following PAP treatment (Figure 1). After treatment with 0.23 mM PAP for 24 h, the inhibition rate of H1299 cells was 11.79%. When the concentration of PAP increased to 0.92 mM, the inhibition rate was 67.03% after 24 h treatment. In addition, the inhibition rate of PAP on H1299 cells was also positively correlated with treatment time and the inhibition rate of H1299 cells reached 95% after treatment with 0.92 mM of PAP for 72 h. The inhibitory effect of PAP was significant after 48 h. When the concentration of PAP was between 0.23 and 0.46 mg/mL, significant inhibition on H1299 cells was achieved by increasing the treatment time. The IC_50_ of PAP on H1299 cell proliferation at 24 h, 48 h and 72 h were 0.69 mM, 0.38 mM and 0.27 mM, respectively. Furthermore, PAP has almost no cytotoxic effects on the fibroblast NIH-3T3 cells (data was not shown). Similarly, the marine active peptides Hem and Dol were reported to be cytotoxic to H1299 cells after conjugation with universal BB agonist in a dose-dependent manner (with IC_50_ values of 15 and 25 nM, respectively) [21]. Yu et al. [11] reported that the pentapeptide CSP (ILYMP, with an IC_50_ of 11.25 mM at 72 h) isolated from *Cyclina sinensis* had inhibitory activity against DU-145 cells in a dose-dependent manner. Wu et al. [12] demonstrated that the pentapeptide AAP-H (YVPGP, with IC_50_ values of 9.605 mM, 7.910 mM, and 2.298 mM at 24 h, 48 h, and 72 h, respectively), purified from the sea anemone *Anthopleura anjunae*, also induced apoptosis in a dose- and time-dependent manner. In conclusion, PAP inhibited the proliferation of H1299 cells in a dose- and time-dependent manner, and PAP concentrations below 0.92 mM had no obvious cytotoxicity to the normal cells.

### 2.2. Morphological Observations

#### 2.2.1. Inverted Microscope Observations

**Figure 2:**
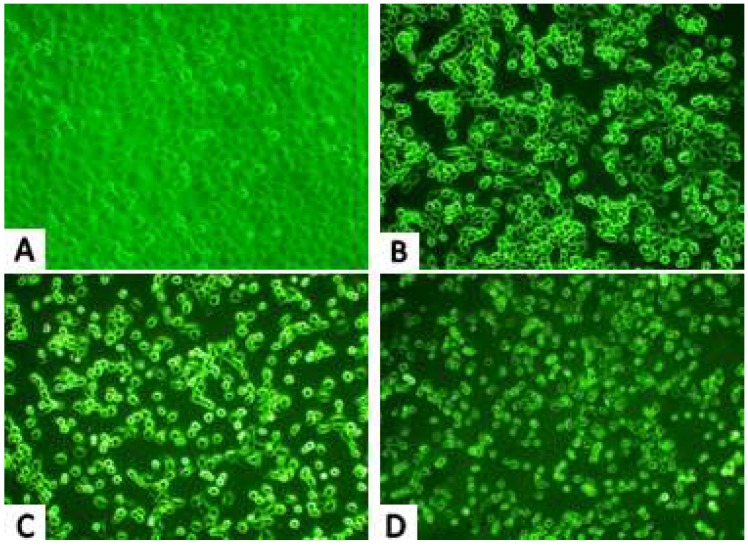
Morphological observation by inverted microscopy (× 200). H1299 cells were untreated (**A**) or treated with 0.23 mM PAP (**B**), 0.46 mM PAP (**C**) and 0.92 mM PAP (**D**). Each experiment was performed in triplicate and the cells exhibited similar morphological features.

Viewing the treated cells with an inverted microscope revealed visible damage to H1299 cells caused by PAP, which was enhanced with increasing of PAP concentrations. As shown in Figure 2, the control cells (Figure 2A) adhered to the bottom of the cell culture flasks and the cells grew tightly. When the cells were treated with 0.23 mM PAP, the cells were mostly rounded and dispersed (Figure 2B). When the PAP concentration reached 0.46 mM (Figure 2C), a small number of cells exhibited an irregular shape, while most cells appeared round and bright. When the PAP concentration reached 0.92 mM (Figure 2D), the treated cells became smaller and were longer stuck to the bottle but floated.

#### 2.2.2. AO/EB Fluorescence Staining Results

**Figure 3:**
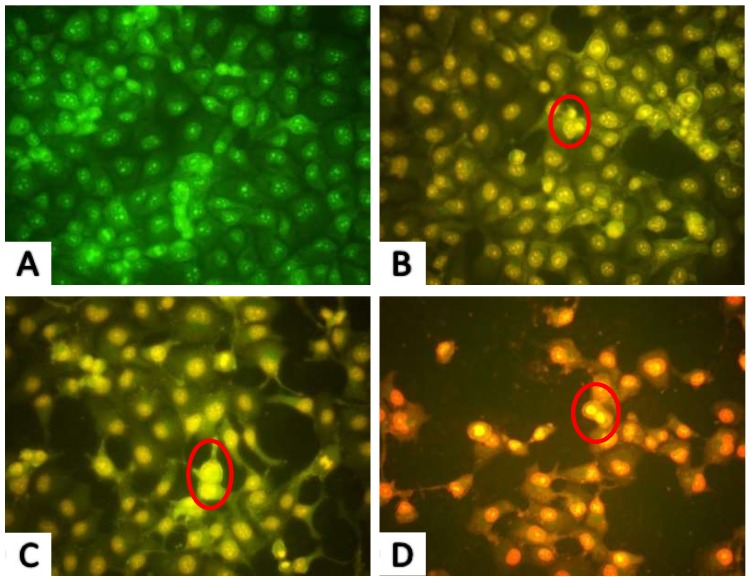
Morphological observation by Acridine orange/ethidium bromide (AO/EB) staining (× 200). H1299 cells were treated with PAP at 0 Mm (**A**), 0.23 mM (**B**), 0.46 mM (**C**), and 0.92 mM (**D**) for 24 h. The red circles in Figure 3B,C indicate early apoptotic cells, while the red circle in Figure 3D indicates late apoptotic cells. Each experiment was performed in triplicate and the cells exhibited similar morphological features.

Acridine orange/ethidium bromide (AO/EB) staining is commonly used for cell morphology and cell cycle analysis. Before the apoptotic rate was calculated by Annexin V-FITC/PI Apoptosis Detection Kit, AO/EB fluorescence staining was used to provide an indication of apoptosis following drug treatment, which can help to determine the appropriate dose and timing of drug intervention. Nuclear chromatin was condensed and distributed along the nuclear membrane in early apoptotic cells. Subsequently, the chromatin further condensed to form apoptotic bodies and the cells entered late apoptosis. The cells in the control group had intact nuclei with uniform green fluorescence and clear cell boundaries observed (Figure 3A). Cells with early apoptotic cell nuclei exhibited yellow-green fluorescence following treatment with 0.23 and 0.46 mM PAP for 24 h, while late-stage apoptotic cells with concentrated and asymmetrically localized nuclear and unclear cyto-membranes were also observed. As the PAP concentration increased to 0.92 mM, apoptotic bodies formed by chromatin condensation or cleavage and the number of late apoptotic cells increased, with necrotic cells showing uneven orange-red fluorescence also observed (Figure 3D). The AO/EB staining results also revealed that the apoptotic characteristics of H1299 cells caused by PAP treatment occurred in a dose-dependent manner.

### 2.3. Cell Apoptosis Analysis

**Figure 4:**
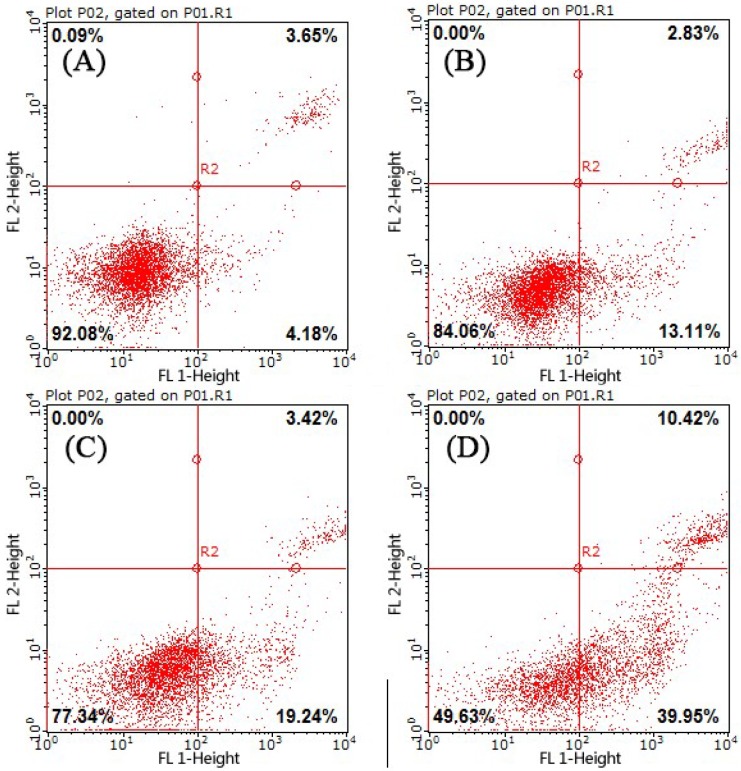
The apoptotic effect of PAP on H1299 cells as determined by flow cytometry. The percentages of early apoptotic cells were 4.18% in the blank control cells (**A**); 13.11% in the 0.23 mM PAP-treated cells (**B**); 19.24% in the 0.46 mM PAP-treated cells (**C**); and 39.95% in the 0.92 mM PAP-treated cells (**D**). One representative apoptosis analysis of three independent experiments was presented.

In the early stages of apoptosis, phosphatidylserine (PS) flips to the surface of the cell membrane and annexin-V is able to bind PS with high affinity. In addition, propidium iodide (PI) can penetrate the incomplete cell membrane to stain the nucleus red, which distinguishes between late apoptotic cells and necrotic cells. The Annexin V-FITC/PI Apoptosis Detection Kit can quantitatively measure the early apoptotic status of cells. Flow cytometry was used to quantitatively detect the apoptosis rate of PAP-treated H1299 cells, which is shown in Figure 4, where the upper left quadrant represents necrotic cells, the lower left quadrant represents normal living cells, the upper right quadrant represents late apoptotic cells, and the lower right quadrant represents early apoptotic cells. After 24 h of treatment with PAP, Annexin V-FITC/PI staining showed that PAP increased apoptosis in H1299 cells in a dose-dependent manner. As shown in Figure 4, the rates of early and late apoptotic stages in H1299 cells without PAP treatment were 4.18% and 3.65%, respectively, with 92.08% of the cells appearing healthy. As the concentration of PAP increased, the number of late apoptotic cells increased from 2.83% to 10.42% and the percentage of cells in the early stages of apoptosis increased dramatically from 13.11% to 39.95%.

### 2.4. Effects of PAP on the Cell Cycle Distribution of H1299 Cells

**Figure 5:**
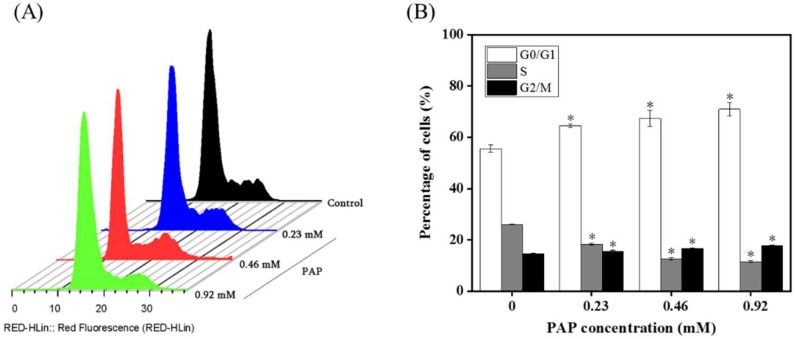
(**A**) Effects of PAP on the cell cycle progression of H1299 cell lines. H1299 cells were treated with PAP at 0, 0.23, 0.46, and 0.92 mM for 24 h. Flow cytometry was used to define the cell cycle distribution in comparison with controls. (**B**) Percentages of H1299 cells in the G0/G1, S and G2/M phases. All data are presented as the mean ± standard deviation (SD) of three experiments. (*) Results are significantly different from the control (*p* < 0.05).

The cell cycle is the series of events that a cell undergoes from the completion of one division phase to the end of the next. In general, the cell cycle can be divided into interphase (phase I) and mitosis (M phase) and the DNA content of cells is different in different stages. PI, a DNA-binding fluorescent dye, exhibits a fluorescence intensity proportional to the amount of DNA bound to intracellular DNA. Therefore, the distribution of DNA in each phase of the cell cycle is directly reflected by flow cytometry and the proportions of cells in the G0/G1 phase, S phase and G2/M phase can be accurately quantified. In the present study, we determined the cell cycle distribution of H1299 cells treated with PAP. The first peak in Figure 5A represents cells in the G0/G1 phase, the second peak represents cells in the G2/M phase and the valley is cells in the S phase. The proportion of cells in each phase is shown in Figure 5B. After treatment with PAP at 0.23, 0.46 and 0.92 mg/mL for 24 h, the proportion of cells in the S phase in H1299 cells significantly increased from 12.28% to 21.79%. Moreover, there was a decreased proportion of H1299 cells in the S phase (from 42.29% to 16.87%). This result indicated that PAP induced G2/M phase arrest in a dose-dependent manner to arrest the proliferation of tumor cells. Similarly, Huang [22] reported that the oligopeptide (Gln-Pro-Lys, SIO) from *Sepia* ink significantly inhibited the S phase of PC-3 cells and the number of cells in the G0/G1 phase showed a similar increase. SIO arrested PC-3 cells in the G0/G1 phase in a dose-dependent manner. Meanwhile, Yang et al. [23] showed that DU-145 cells treated with a tetrapeptide (Asp-Trp-Pro-His, PROI-1) had a significantly reduced number of cells in the S phase, leading to induction of G2/M phase arrest and apoptosis.

### 2.5. Western Blotting Results

Apoptosis is a process of programmed cell death that is induced by specific signals produced by various physiological or pathological stimuli. Therefore, the analysis of signaling factors involved in the regulation of apoptosis is useful for elucidating the mechanism of action of the anti-proliferative effects of drugs. To further verify the effects of PAP on H1299 cells and explain the underlying mechanisms, western blotting was used to investigate the expression of apoptosis-related proteins in treated H1299 cells.

**Figure 6:**
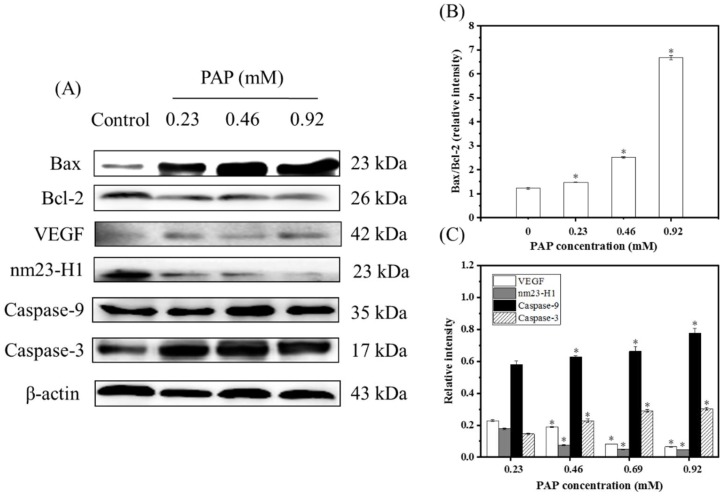
(**A**) Expression of Bax, Bcl-2, VEGF, nm23-H1, Caspase-9, Caspase-3 proteins in H1299 cells treated with different concentrations of PAP (0, 0.23, 0.46 and 0.92 mM) for 24 h. (**B**) The Bax/Bcl-2 ratio expressed in H1299 cells treated with PAP as a function of PAP concentrations for 24 h, where Bax and Bcl-2 were apoptosis-associated. (**C**) VEGF, nm23-H1, Caspase-9, Caspase-3 expression in H1299 cells treated with PAP for 24 h. The blots were detected with β-actin antibody to determine equal sample loading. Each experiment was performed in triplicate. (*) Results are significantly different from the control (*p* < 0.05).

Apoptosis is regulated by key regulatory proteins of the Bcl-2 family. Most Bcl-2 family proteins play a role at the mitochondrial level, and excessive apoptosis caused by abnormal changes in the levels of Bcl-2 proteins is a key step in tumor progression [24,25]. Up-regulation of *Bcl-2* gene expression is conducive to the inhibition of apoptosis and anti-apoptotic effect [26]. The *Bax* gene also belongs to the *Bcl-2* gene family and Bax forms a heterodimer with Bcl-2, which inhibits Bcl-2 and promotes apoptosis [27]. A previous study found that the proportional relationship between Bax/Bcl-2 proteins is a key factor in determining the inhibition of apoptosis [24,25]. As shown in Figure 6, PAP showed pro-apoptotic activity against H1299 cells with the Bax/Bcl-2 expression ratios of 1.47%, 2.52% and 6.67%, when the cells treated with 0.23, 0.69 and 0.92 mg/mL of PAP, respectively. Huang [22] reported that SIO induced the death of prostate cancer cells (DU-145, PC-3, and LNCaP) and showed an increase in the Bax/Bcl-2 ratio. Furthermore, Wan et al. [28] found that an oligopeptide (KIKAVLKVLTT) derived from melittin induced the death of thyroid cancer TT cells via upregulation of Bax and down-regulation of Bcl-2. Our results also indicated that PAP induced apoptosis of H1299 cells by up-regulating the Bax/Bcl-2 ratio, which was consistent with previous studies.

Caspase proteins are proteolytic enzymes that play important roles in programmed cell death. They can be further subdivided into the following two types according to their function in apoptosis: initiator (caspases-9) and effector (caspases-3) caspases [29]. Caspase-9 is activated upon cytochrome c release and acts to activate effector caspase and the pro-apoptotic protein Bid, which ensures that cells with an apoptosome will die without effector caspase. Furthermore, caspase-3 is a major executor of apoptotic death, which makes cell death more effective. PAP significantly up-regulated the expression of caspase-9 and caspase-3. When the PAP concentration ranged from 0 to 0.92 mg/mL, the relative intensities with caspase-9 and caspase-3 increased from 0.58 to 0.78 and 0.15 to 0.30, respectively. This indicated that PAP treatment causes a cascade of reactions in H1299 cells leading to apoptosis.

Nm23-H1 is widely used as an important indictor to determine tumor metastasis. The nm23-H1 protein is highly similar to the α-chain amino acid sequence of the nucleoside diphosphate kinase (NDPK) and has the same activity. It plays a role in cell growth and tumor metastasis by catalyzing the conversion of guanosine triphosphate GTP to guanosine diphosphate (GDP), participating in the polymerization and disintegrating tubulin or by regulating GDP synthesis to participate in G-protein-regulated transmembrane signaling [29]. For example, L9981 cells transfected with nm23-H1 cDNA showed lower expression of nm23-H1 than untransfected cells, and tumor proliferation and invasiveness decreased at the same time [30]. The results of this experiment also showed that the lower the expression of nm23-H1, the lower the degree of tumor differentiation, and the results of the apoptosis experiment were consistent with the western blotting results [31].

VEGF is an essential angiogenic growth factor. High levels of VEGF were found in approximately one-third of NSCLC [32]. VEGF promotes angiogenesis, regulates the apoptosis of vascular endothelial cells and alveolar epithelial cells and increases the permeability of blood vessels, which leads to sustained tumor growth. For example, Xu [33] studied the inhibitory effect of MUC1 on NSCLC cells and the results of western blotting indicated that MUC1 significantly reduced the levels of VEGF and VEGF-C proteins, indicating that MUC1 has an anti-angiogenic effect and can be used as a potential treatment for NSCLC. In the present study, the expression level of VEGF decreased significantly when the PAP concentration increased, indicating that PAP could inhibit tumor angiogenesis and inhibit tumor growth.

## 3. Materials and Methods

### 3.1. Materials and Reagents

**Figure 7:**
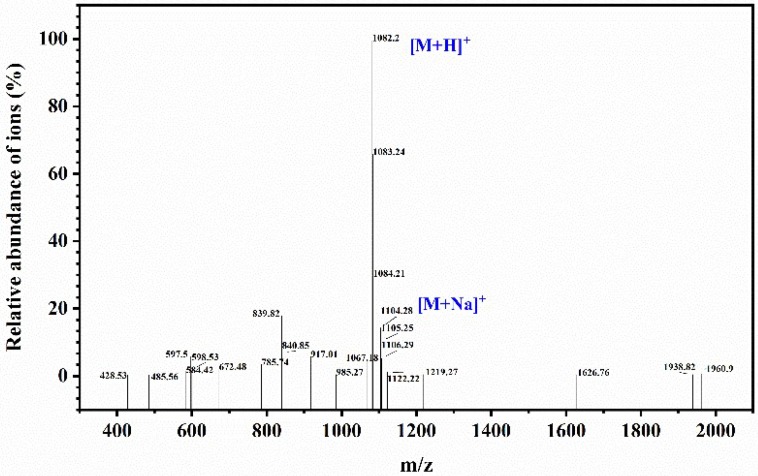
Mass spectrogram of PAP and the sequence of PAP identified as IEPGTVGMMF with a molecular weight of 1081.20 Da.

In a previous study, the peptide PAP (IEPGTVGMMF) was prepared by our laboratory and preserved at −20°C until further use (Figure 7) [20]. The H1299 cell lines and NIH-3T3 cell lines were stored in our laboratory. Cell Counting Kit-8 (CCK-8) and Cell Cycle and Annexin V-FITC/PI Apoptosis Detection Kit were purchased from BestBio Biotechnology (Shanghai, China). The BCA protein detection kit was purchased from Jiancheng Bio-Technology Co., Ltd. (Nanjing, China). Antibodies against β-actin (cat. no. TA-09), Bax (cat. no. 2772S), Bcl-2 (cat. no. 2872S), nm23-H1 (cat. no. bs-1066P), VEGF (cat. no. AF1309), Caspase-3 (cat. no. 9662S), and Caspase-9 (cat. no. 70R-11636) were purchased from ZSGB Biotechnology (Beijing, China). All other reagents used in this study were of analytical grade.

### 3.2. Detection of Anti-Proliferation Activity Using CCK-8

The CCK-8 detection kit is a simple and accurate method that is widely used in cell proliferation analysis [22]. The following steps were performed, according to the method of Huang et al. [22] with slight modifications. H1299 cells were adjusted to approximately 1 × 10^4^ cells/100 µL/well in a 96-well tray and incubated overnight in a 5% CO_2_ incubator (Forma 3111 CO_2_ incubator, Thermo Forma, Waltham, MA, USA) at 37 °C. The cells were then treated with PAP at final concentrations of 0, 0.23, 0.46, 0.69 and 0.92 mM for 24, 48 or 72 h. After the treatment was completed, the culture medium was removed, and 90 µL of phosphate-buffered saline (PBS) and 10 µL CCK-8 were added to each well. Incubation was carried out for 4 h under conventional conditions in a CO_2_ incubator and the optical density (OD) value was measured with an automatic microplate reader (SpectraMax M2, Molecular Devices, Sunnyvale, CA, USA) at a detection wavelength of 450 nm. The following equation was used to calculate the relative inhibition rate of cell proliferation:

Relative inhibition rate (%) = (1 − (OD_treated_/OD_control_)) × 100%.

### 3.3. Cell Morphology Observation Using an Inverted Microscope

H1299 cells (approximately 1 × 10^5^ cells/well) were seeded in a 6-well flat-bottom plate and incubated for 24 h in a 5% CO_2_ incubator at 37 °C. The culture medium was then removed and the cells were treated with PAP at a final concentration of 0, 0.23, 0.46 and 0.92 mM. After incubation for another 24 h, morphological changes in the cells were observed using a CKX4 inverted microscope (OLYMPUS, Tokyo, Japan).

### 3.4. Cell Morphological Analysis by AO/EB Staining

To observe the morphological characteristics of H1299 cells at different stages of apoptosis, cells were stained using AO/EB fluorescence staining as described by Huang et al. [34], with slight modifications. Cleaned coverslips were placed in a 6-well plate (approximately 1 × 10^5^ cells/well) and H1299 cells were seeded as previously described. After the cells were attached, the cells were treated with final concentrations of 0, 0.23, 0.46 and 0.92 mM PAP for 24 h. The coverslips were washed 2 to 3 times with PBS (pH 7.2) and fixed in 95% ethanol for 30 min. Drops containing 50 μL of PBS and 6 μL of AO/EB mixture (0.1 mg/mL AO and EB in PBS, pH = 7.2) were placed on microscope slides, and the side of the coverslip to which the cells had adhered was placed in contact with the AO/EB droplet. Cell morphology was observed by a BX41 fluorescence microscope (OLYMPUS, Tokyo, Japan) and photographed.

### 3.5. Cell Apoptosis Analysis Using Annexin V FITC/PI

To confirm PAP-induced apoptosis of H1299 cells, flow cytometry was performed as described by Yoon et al. [35]. H1299 cells were seeded in 25-mL culture bottles (approximately 1 × 10^5^ cells/mL). After 24 h incubation, cells were treated with different final concentrations (0, 0.23, 0.46 and 0.92 mM) of PAP and cultured for 24 h. Cells were then harvested following treatment and digested with trypsin. The suspended cells were then collected by centrifugation (1000 rpm, 5 min) and were stained with Annexin V-FITC and PI using the Annexin V-FITC Apoptosis Detection Kit. Finally, apoptosis was immediately detected by flow cytometry (Becton Dickinson, NJ, USA).

### 3.6. Cell Cycle Analysis by Propidium Iodide Staining

To investigate whether PAP controls the cell cycle to achieve apoptosis, flow cytometry was used to measure the different cell cycle phases of H1299 cells, following the method described by Li et al. [36]. H1299 cells in the logarithmic growth phase were inoculated into a 6-well plate and the cells were cultured for 24 h until they had completely adhered to the bottom of the plate. Different concentrations of PAP (0, 0.23, 0.46 and 0.92 mM) were added to the cells and they were incubated for 24 h. Then cells were trypsinized, harvested and washed twice with pre-cooled PBS. Briefly, RNase A was added to the cells obtained by centrifugation and the mixture was incubated at 37 °C for 30 min. Finally, 350 μL of PI was added, mixed and incubated at 4 °C for 5 min in the dark. The solution was subsequently filtered with a 200 mesh sieve and a cell cycle curve was obtained using flow cytometry.

### 3.7. Detection of Protein Expression by Western Blotting

To confirm the apoptotic effects of PAP on H1299 cells, we performed western blotting according to the method described by Peng et al. [37]. H1299 cells were seeded in a 6-well plate (approximately 1 × 10^5^ cells/mL) and treated with different final concentrations of PAP (0, 0.23, 0.46 and 0.92 mM). After treatment with PAP for 24 h, cells were harvested and lysed in RIPA lysis buffer. The extracted proteins were quantified by the bicinchoninic acid (BCA) total protein assay kit. SDS-PAGE was used to separate proteins, which were subsequently blotted onto PVDF membranes (Millipore, Billerica, MA, USA). Five percentage of skim milk was used as a blocking solution for 1 h and incubated overnight with the primary antibodies (Bcl-2, Bax, nm23-H1, VEGF, Caspase-3, and Caspase-9) at 4 °C. After washing twice with TBST (10 mL Tris-buffered saline with 20% Tween-20) and once with TBS (10 mL Tris-Buffered saline), membranes were incubated with the secondary antibodies for 2 h. Finally, membranes were washed as noted above and combined with enhanced chemiluminescence (ECL) reagents and images were captured using an Alpha FluorChem FC3 imaging system (ProteinSimple, San Jose, CA, USA). β-actin was used as an internal control. Image J 1.38 software (NIH, Bethesda, MD, USA) was used to quantify and record the OD.

### 3.8. Statistical Analysis

All experimental data are expressed as the mean ± standard deviation ($\bar{x}$ ± *s*, n = 3), and were analyzed using SPSS software version 24.0 (SPSS Inc., Chicago, IL, USA). Statistical significance of the data was compared using one-way analysis of variance (ANOVA). The least significant difference (LSD) was used for post hoc multiple comparisons, and *p* < 0.05 indicates a statistically significant difference.

## 4. Conclusions

In the present study, PAP (Ile-Glu-Pro-Gly-Thr-Val-Gly-Met-Met-Phe, IEPGTVGMMF) that was purified from an enzymatic hydrolysate of *Perinereis aibuhitensis* showed anti-cancer activity toward H1299 cells. The CCK-8 results showed that PAP inhibited the proliferation of H1299 cells in a time- and dose-dependent manner. The apoptotic status of the cells was also observed with an inverted microscope and AO/EB staining. The results of flow cytometry showed that PAP could induce apoptosis of H1299 cells and the apoptosis rate increased with increasing drug dosage. Therefore, PAP may inhibit the growth of malignant lung cancer cells by inducing G0/G1 phase arrest and tumor cell apoptosis. Furthermore, the results of western blotting showed that the expression of nm23-H1 and VEGF protein decreased in a dose-dependent manner, while the ratio of pro-apoptotic proteins and anti-apoptotic proteins, apoptosis-related caspase proteins increased in a dose-dependent manner. In conclusion, our results indicated that PAP has the potential to be used as the functional or adjuvant food for the prevention or treatment of human NSCLC in the future. However, studies on the structure–activity relationship of PAP and studies on the anticancer activity in vivo of this peptide need to be performed.
